# Microbial Diversity of Browning Peninsula, Eastern Antarctica Revealed Using Molecular and Cultivation Methods

**DOI:** 10.3389/fmicb.2017.00591

**Published:** 2017-04-07

**Authors:** Sarita Pudasaini, John Wilson, Mukan Ji, Josie van Dorst, Ian Snape, Anne S. Palmer, Brendan P. Burns, Belinda C. Ferrari

**Affiliations:** ^1^School of Biotechnology and Biomolecular Sciences, University of New South WalesKensington, NSW, Australia; ^2^Australian Antarctic Division, Department of Sustainability, Environment, Water, Population and CommunitiesKingston, TAS, Australia

**Keywords:** antarctic soil, frost boils, bacterial diversity, fungal diversity, cultivation

## Abstract

Browning Peninsula is an ice-free polar desert situated in the Windmill Islands, Eastern Antarctica. The entire site is described as a barren landscape, comprised of frost boils with soils dominated by microbial life. In this study, we explored the microbial diversity and edaphic drivers of community structure across this site using traditional cultivation methods, a novel approach the soil substrate membrane system (SSMS), and culture-independent 454-tag pyrosequencing. The measured soil environmental and microphysical factors of chlorine, phosphate, aspect and elevation were found to be significant drivers of the bacterial community, while none of the soil parameters analyzed were significantly correlated to the fungal community. Overall, Browning Peninsula soil harbored a distinctive microbial community in comparison to other Antarctic soils comprised of a unique bacterial diversity and extremely limited fungal diversity. Tag pyrosequencing data revealed the bacterial community to be dominated by Actinobacteria (36%), followed by Chloroflexi (18%), Cyanobacteria (14%), and Proteobacteria (10%). For fungi, Ascomycota (97%) dominated the soil microbiome, followed by Basidiomycota. As expected the diversity recovered from culture-based techniques was lower than that detected using tag sequencing. However, in the SSMS enrichments, that mimic the natural conditions for cultivating oligophilic “k-selected” bacteria, a larger proportion of rare bacterial taxa (15%), such as *Blastococcus, Devosia, Herbaspirillum, Propionibacterium* and *Methylocella* and fungal (11%) taxa, such as *Nigrospora, Exophiala, Hortaea*, and *Penidiella* were recovered at the genus level. At phylum level, a comparison of OTU's showed that the SSMS shared 21% of Acidobacteria, 11% of Actinobacteria and 10% of Proteobacteria OTU's with soil. For fungi, the shared OTUs was 4% (Basidiomycota) and <0.5% (Ascomycota). This was the first known attempt to culture microfungi using the SSMS which resulted in an increase in diversity from 14 to 57 microfungi OTUs compared to standard cultivation. Furthermore, the SSMS offers the opportunity to retrieve a greater diversity of bacterial and fungal taxa for future exploitation.

## Introduction

**Figure 1:**
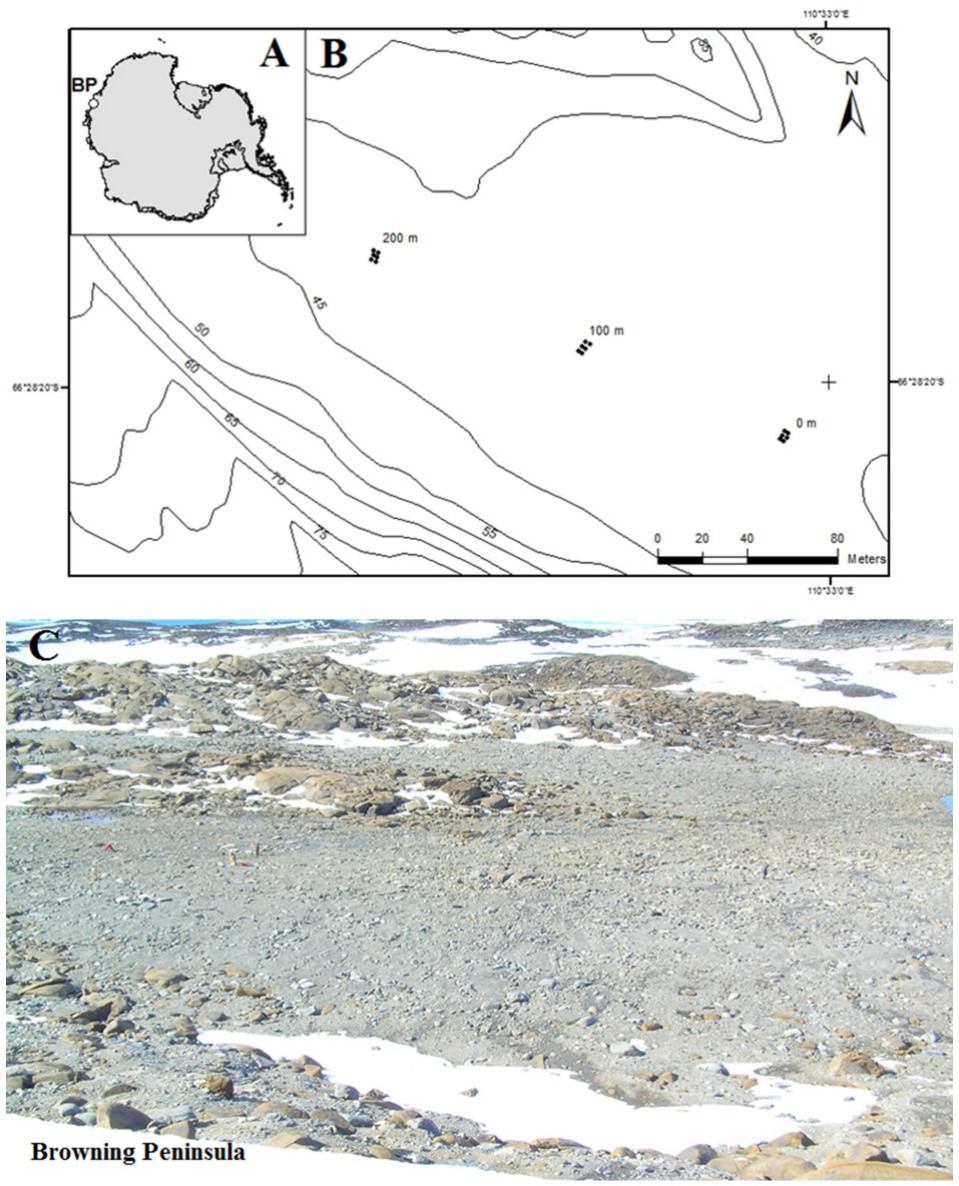
**(A)** Location of Browning Peninsula, Windmill Islands, Eastern Antarctica. **(B)** The spatially explicit design of sampling along three parallel transects, each 2 m apart. The sampling points were taken at 0, 2, 100, 102, 200, and 202 m distances along all three transects. **(C)** The photograph depicts a close up of Browning Peninsula.

Browning Peninsula is a remote and understudied area at the Southern end of the Windmill Islands, Eastern Antarctica. It lies 20 km away from the well-studied Casey station (Chong et al., 2009), and is described as an ice-free desert landscape comprised of barren rocks (Figure 1) with low diversity of invertebrates and vascular plant life (Azmi and Seppelt, 1998; Stewart et al., 2011). The mean annual temperature is −9.3°C with the average temperature rising above freezing point (+0.2°C) only in January (Beyer and Bolter, 2000), and annual precipitation is approximately 176 mm falling primarily as snow (Beyer and Bolter, 2000). The entire valley is described as a polar desert, comprised of frost boils ranging from 2 to 10 m in diameter (Stewart et al., 2011; Ferrari et al., 2015).

Frost boils are upheavals of mud that occur through frost heave and freeze-thaw processes common in permafrost regions (Walker et al., 2004). In the Arctic, frost boils have been reported to exhibit a higher accumulation of organic materials within undisturbed inter-boil areas, leading to a systematic variation of vegetation growth (Walker et al., 2004). As a result of frost heave, soil biomass can increase from approximately 183 to 831 g m^−2^ and this effect is highest within frost boils that are comprised of fine grain sediments. Due to the extreme temperatures, limited water availability, reduced growing season, unstable substrates and severe local climatic conditions (Melick et al., 1994), no vegetation or bryophytes have been observed on Browning Peninsula (Stewart et al., 2011) and the effect on other biomass is unknown. However, the soil organic content has been found to be lower than that in Arctic soils, with Browning Peninsula soils classified as nutrient-poor and low in total carbon, nitrogen and moisture (Ferrari et al., 2015).

Cold-adapted microbes have great potential for the biotechnological industry. The biomass produced via the cultivation of microbes from extreme environments has led to the discovery of extracellular substances with antibacterial and/or antifungal properties (Gesheva, 2010; Gesheva and Negoita, 2011; Tomova et al., 2015). Additionally, the production of surfactants (glycolipids), the ability to grow on paraffin and naphthalene (Gesheva and Negoita, 2011) and their resistance to heavy metals (Tomova et al., 2015), highlights the potential for cold-adapted microbes to emulsify contaminants, such as hydrocarbons. Continued isolation of rare polar species could provide access to further strains for biotechnological applications, such as bioremediation and discovery of novel bioactive compounds, such as antibiotics (Tosi et al., 2010; Tytgat et al., 2014).

It is well established that a very small percentage of bacterial and fungal species are able to grow under traditional culture conditions (Handelsman, 2004; Ferrari et al., 2008, 2011). It is now known that the application of diverse sample dilutions, culture media, pH, incubation temperature and atmospheres, increases the metabolic diversity of species that can be cultivated from a single sample (Donachie et al., 2007). Novel methods have also been developed to increase the microbial biota representation under culture conditions and as a result select for a greater range of oligotroph. Some extend incubation times (Davis et al., 2005), or use dilute media (Ferrari et al., 2011), while others mimic the natural environment, for example implementing a diffusion chamber (Ferrari et al., 2005; Bollmann et al., 2007, 2010) or an *in-situ* trap (Gavrish et al., 2008). The excessive supply of nutrients in the artificial media often selects for fast-growing fungi and bacteria and to address this problem in soil, a simulated environment that mimics the natural soil environment called the soil slurry membrane system (SSMS) was developed (Ferrari et al., 2005). The diffusion chamber approach relies on the use of soil as the substrate for growth and has been found to enrich for members from a diversity of cultured and uncultured bacterial phyla, including the Saccharibacteria, better known as Candidate division TM7 (van Dorst et al., 2016). While the approach has been adopted to enrich for bacteria, the suitability of the method to select for fungi is not yet known.

Modern molecular phylogenetic and high throughput DNA sequencing have provided a comprehensive culture-independent analysis of soil microbial diversity (Margulies et al., 2005; Cowan et al., 2014), thereby accelerating the rate of gene discovery and giving rise to many applications in biotechnology (Handelsman, 2004; Jansson and Tas, 2014). Recently, NGS tools have been used to uncover bacterial (Tytgat et al., 2014; van Dorst et al., 2014; Ji et al., 2015) and fungal diversity (Kochkina et al., 2012; Ji et al., 2015) from Antarctic soil sources. This not only revealed previously unclassified bacterial diversity but also increased the size of microbial diversity datasets, improving access to vast microbial communities within a range of biospheres (Tytgat et al., 2014; Ji et al., 2015).

Limited studies have been conducted on Browning Peninsula. Studies to date have compared bacterial biomass between soil types (with and without penguin colonies) (Roser et al., 1993), isolated soil microfungi (Azmi and Seppelt, 1998), surveyed bacterial diversity using denaturing gradient gel electrophoresis (Chong et al., 2009) and examined the soil physical, chemical and microbial properties (Stewart et al., 2011). Most recently, analysis of environmental drivers of polar soil diversity and structure across the poles showed bacterial and fungal communities are driven primarily by soil fertility and pH (Siciliano et al., 2014). Microbial community connectivity within soils at Browning Peninsula was found to be highly fragmented and this fragmentation or dis-connectivity mirrored the fragmented nature of the frost boil landscape (Ferrari et al., 2015).

Several microbial ecology studies have compared the recovery of bacterial and fungal diversity using culture-dependent and culture-independent techniques, with only minor overlaps in microbial taxa observed (Donachie et al., 2007; Kochkina et al., 2012; Tytgat et al., 2014; Stefani et al., 2015). In these cases, traditional cultivation methods using standard cultivation media and different incubation temperatures were used and selected primarily for R-strategists (Watve et al., 2000; Ferrari et al., 2011). In this study, our aim was to use a traditional cultivation, novel micro-cultivation (SSMS), and tag pyrosequencing to uncover the total bacterial and fungal diversity present within soils of Browning Peninsula. To our knowledge, this research is the first report of enriching fungi, particularly microfungi using the SSMS.

## Materials and methods

### Site description and sampling

The soil was sampled from browning Peninsula (Figure 1) in 2005 as a part of larger biodiversity project (Siciliano et al., 2014). Due to limitations of sampling site, such as erratic boulders, frost wedges, melt ponds and unstable slopes, the spatially explicit design uses three parallel transects which together provide a reasonable sample representation across the site (Siciliano et al., 2014). A total of 18 samples was selected for this investigation, from three transects, 2 m apart, with samples taken at variable lag distances of 0, 2, 100, 102, 200, and 202 m (Figure 1C). The soil samples (1 kg) were taken from top 10 cm of the soil profile, aseptically sieved and stored at −80°C until analyzed. Samples were taken from a number of different frost boils and all frost boils that were sampled from were given arbitrary number from 1 to 42.

### Physical and chemical analysis of soil samples

Comprehensive physical and chemical properties of all 18 soil samples were obtained using standard approaches (van Dorst et al., 2014). Briefly, variables, such as location, elevation, slope and aspect were obtained using a combination of global positioning system (GPS), geographic information system (GIS) and site digital elevation models. Physical variables, such as grain size was measured by sieving soil particles larger than 2 mm and separating other particles based on grain size into mud (<63 μm) and sand (63–2000 μm). Chemical analysis, such as conductivity, pH, ${\text{NO}}_{2}^{-}$, ${\text{NO}}_{3}^{-}$, ${\text{PO}}_{4}^{3-}$ and ${\text{SO}}_{4}^{-}$ was obtained using 1 in 5 dilution of soil on the dry mass basis (mg kg^−1^). Total carbon (TC), total phosphorus (TP), and total nitrogen (TN) were measured by combustion and NDIR gas analysis (Rayment and Lyons, 2011). The measurement of other elements concentration, such as SO_3_,P_2_O_5_, and Cl were determined using X-Ray Fluorescence (XFR) analysis.

### Soil slurry membrane system (SSMS) micro-cultivation

The SSMS setup was performed following the protocol described by Ferrari et al. (2008) with slight modifications. Briefly, approximately 3–5 g of soil was placed into a sterile Tissue Culture Insert (TCI) (Merck Millipore; MA, USA) and the soil slurry was prepared by adding a few drops of water into the TCI and mixing well using a vortex. An inoculum was prepared by adding 1 g of the same soil to 10 ml of water and mixing well using vortex. The inoculum was then allowed to stand for 5 min to settle heavy particles and 100 μl of inoculum was filtered onto a 0.22 μm, 25 mm PolyCarbonate (PC) membrane (Merck Millipore; MA, USA) using filtration manifold. The PC membrane was then placed on top of pre-prepared TCI, within a six-well plate and incubated at 8°C for 21 days.

### Fluorescent staining of enriched communities

After 21 days of incubation, the presence of adequate growth of the micro-colonies on SSMS membranes was confirmed using epi-fluorescence microscopy following SYBR green staining (Invitrogen; Paisley UK). A quarter slice of a growth membrane was cut using a sterile scalpel blade and forceps and a thin layer of heated 1% agarose was then poured into a glass slide. The slice was inverted onto the agarose and allowed to dry at 30°C for 15 min. The growth membrane was taken from the slide and a drop of Vectashield mounting medium containing DAPI (Vector laboratories Inc, Burlingame) was added onto the remaining agarose. Subsequently, 10 μl of 1:100 dilutions of SYBR green was added to the agar. To stain fungi, 10 μl of 2X Calcofluor white stain (Sigma-Aldrich, Australia) was also added to the agarose and a coverslip applied. The microbial growth was then visualized using an Olympus BX61 microscope (Olympus, North Ryde Australia), with appropriate filters for green (Bacteria) and UV (fungi) fluorescence.

### DNA extraction from growth membranes

The *prep*GEM DNA extraction protocol was used as described in Ferrari et al. (2008) with some modifications. Firstly a quarter slice of each growth membrane was placed in a PCR tube with 99 μl of 1X Buffer (diluted from 10X) and 1 μl of *prep*GEM enzyme. Samples were mixed and placed in a thermocycler (Bioteke corporation), following the program of 37°C for 15 min, 75°C for 15 min, and 95°C for 15 min. The microfuge tube was centrifuged at 14,000 rpm for 3 min. The membrane slice was then removed with the help of sterile tweezers and 80 μl of DNA lysate was transferred to a new PCR tube. The genomic DNA was quantified using Quanti-iT™ Picogreen® dsDNA reagent (Invitrogen, Paisley UK) following manufacturer's instructions and stored at −20°C until used.

### Recovery of total genomic DNA from soil

Genomic DNA was extracted in triplicate from the soil samples using the MP FastDNA™ SPIN Kit for soil (MP Biomedicals, NSW Australia), as described (van Dorst et al., 2014). The DNA extracts were quantified using Quanti-iT™ Picogreen® dsDNA reagent (Invitrogen, Paisley UK) on a black 96 well plate at an absorbance of 520 nm using a fluorescence plate reader (SpectraMax M3 Multi-Mode Microplate Reader; Molecular Devices, CA). The DNA lysate concentrations produced ranged from 0.21 to 0.37 ng μl^−1^ and were stored at −80°C until used further.

### Tag pyrosequencing of soil and SSMS microbial communities

Bacterial ARISA was performed to allow for the selection of replicates for tag sequencing. In comparison with the metabarcoding approach, ARISA is a cheap and rapid alternative, ideal for ensuring appropriate replication in large microbial ecology studies (van Dorst et al., 2014). All soil DNA was extracted in triplicate and then ARISA was used to evaluate technical replicates and ecological patterns. One of each of the technical replicates was then selected for sequencing. Briefly, ARISA PCR was carried out targeting the bacterial ITS region using the universal primers 1392f; 5′ GYACACACCGCCCGT 3′and 5′ MAX labeled 23Sr 5′GGGTTBCCCCATTCRG3′ (Fisher and Triplett, 1999; Hewson and Fuhrman, 2006). The PCR reaction mix included 0.5 μM of forward (1392) and reverse primer (5′ MAX labeled 23Sr), 1X buffer, 2.5 mM MgCl_2_ (Promega), 0.25 mM of each dNTP (Promega), and 0.8 μg μl^−1^ of BSA (Promega), 1 unit Taq polymerase with addition of water to final reaction volume of 25 μl. The cycling temperature was 94°C for 2 min denaturation temperature followed by 30 cycles of amplification at 94°C for 30 s, 55°C for 30 s, and 72°C for 30 s with a final extension 72°C for 5 min. The fragment separation was performed at Macquarie University on an Applied Bio-system 3730 fragment analyzer (Life technology), which was further interpreted via GENEMAPPER software (Life Technology). The sequences obtained were further filtered by removing the background fluorescence. Fragments were assigned to appropriate bins and final data were transformed into peak area verses sample matrix where the single peak represented single OTU (van Dorst et al., 2014). The samples were then compared for shared and unique bacterial OTUs using the Primer 6 version 6.1.13 and PERMANOVA version 1.0.3 software and a nonmetric multidimensional scaling (nMDS) plot and a cumulative rank abundance was generated using a Bray-Curtis similarity matrix (Clarke and Gorley, 2006; van Dorst et al., 2014).

After confirming a strong correlation between technical replicates using ARISA (van Dorst et al., 2014), a representative gDNA extract from each replicate was sent for barcode tag pyrosequencing to an external facility (MR DNA, Molecular Research Laboratory, TX USA) which was performed on the Roche 454 FLX titanium platform. The research laboratory followed a single step 30 cycle PCR using Hot StartTaq Plus Master Mix Kit (Qiagen, Valencia, CA) with the following conditions: 94°C for 3 min denaturation, followed by 28 cycle of 94°C for 30 s, an annealing temperature of 53°C for 40 s and elongation at 72°C for 1 min with a final extension step at 72°C for 5 min (Dowd et al., 2008). The bacterial universal primers, 28F and 519R were used to amplify the bacterial DNA (Dowd et al., 2008). For fungi, the ITS1 and ITS2 region was targeted with the primers ITS1 and ITS4 (Gardes and Burns, 1993). Following PCR, all amplicons from different samples were mixed in equal concentration and purified using Agencourt Ampure (Agencourt Bioscience Corporation; MA, USA).

### Processing pipeline for tag pyrosequencing data

#### Bacteria

Raw 454 pyrosequenced data were received in the form of a standard flowgram format (sff) file. Flowgrams were processed according to Schloss et al. (2011) using MOTHUR software Version 1.32.1. Initially, bacterial 16S rRNA reads were denoised by the implementation of PyroNoise component ensuring all reads were 200 bp long. This step included removal of the barcode, primer sequences, sequences with homo-polymers longer than 8 bp and retrieving the reverse complement of each sequence (Quince et al., 2011). An alignment of sequences was generated against the SILVA-compatible alignment database (Pruesse et al., 2007) and chimeras and other contaminant sequences were removed (Schloss et al., 2009). The sequences were pre-clustered at 1% to account for 454's titanium instrument error (Huse et al., 2010). Aligned sequences were then clustered into OTUs at 4% divergence (96% similarity) for the best definition of species level (van Dorst et al., 2014). In addition to this, all singletons generated were removed (van Dorst et al., 2014). A sample by OTU abundance matrix was generated using the MOTHUR software pipeline (Schloss et al., 2009) and the taxonomic assignment was determined against the Greengenes database (2013 May version) (Kim et al., 2011).

#### Fungi

Quality filtering was carried out using MOTHUR software Version 1.32.1 in a similar way to Bacteria. Chimeras were removed from the sequence data using UCHIME (Edgar et al., 2011). The sequence grouping was completed by adding the nucleotide sequences of tag barcodes and identified sequences using the SEED platform (Overbeek et al., 2014). The barcode sequences were removed using SEED. The ITS1 region was then detected and extracted with the ITSx (an improved software), as the ITSx tool has the capacity to extract higher proportions of true positives and remove non-ITS sequences from data sets (Bengtsson-Palme et al., 2013). The obtained reads were then clustered together at 97% similarity to closely define species using USEARCH, representative sequences were identified using mafft and singletons were removed (Caporaso et al., 2010). The sequences were then compared against the UNITE fungal ITS database (Koljalg et al., 2005). A sample by OTU abundance matrix was generated using the SEED package (Overbeek et al., 2014).

### Multivariate data analysis

Diversity indices were generated within MOTHUR (Schloss et al., 2009) and utilized as part of the multivariate analysis. The bacterial and fungal OTU sequence datasets were transformed to square root, standardized and (dis) similarity matrix was generated using Bray-Curtis coefficient (Clarke and Gorley, 2006; Anderson et al., 2008). Using a Primer 6 version 6.1.13 and PERMANOVA version 1.0.3 software PCO plots were configured for visualization and overlaid with normalized environmental data using Person correlation (0.5) (Clarke and Gorley, 2006).

### Selection of samples for cultivation

Following analysis of total microbial diversity, six of the 18 soils, were selected for cultivation experiments. The selection was based on the nMDS plot generated and the clustering of bacterial OTU abundances was performed at community similarities of 40% (samples similarity threshold within frost boils). Each represented a major cluster of community and a different frost boils across the sample landscape (Supplementary Figure 2). The matrix generated for the nMDS plot was also used for analysis of similarities (one-way ANOSIM) and factor frost boil was tested for its significance against soil environment, bacterial communities, and fungal communities.

### Multivariate analysis of environmental data: a distance-based linear models (DistLM) analysis

The environmental parameters were combined into a matrix and visualized with a draftmans plot. The right skewed variables, such as elevation, slope, TN, sand, mud, NO_2_, NO_3_, TKP, pH, TC, PO_4_, SO_3_, SO_4_, P_2_O_5_ and conductivity were log transformed then all variables were normalized. The normalized environmental variables were used to create a resemblance matrix based on the Euclidean distance between samples (Supplementary Tables 2, 3). The environmental resemblance matrix was then used for a DistLM analysis in Primer and PERMANOVA (Clarke and Gorley, 2006; Ferrari et al., 2015). The selected criteria included adjusted R^2^ and step-wise procedures.

### Cultivation using artificial media

Fungi and bacteria were isolated by suspending 1 g of soil with 10 ml of ultrapure milliQ water and further diluted to between 10^2^ and 10^4^. Aliquots of 100 μl were transferred onto various media including BG11 (Sigma-Aldrich Castle Hill Australia), NA (Nutrient Agar, Sigma-Aldrich Castle Hill Australia), SCA (Starch Casein Agar- US Biological, Swampscott USA) and RAVAN (Watve et al., 2000) for bacterial isolation. For fungal isolation, PDA (Potato dextrose agar- Sigma-Aldrich Castle Hill Australia), CRBA (Crook Rose Bengal Agar) and MEA (Malt Extract Agar-Sigma-Aldrich Castle Hill Australia) were used. All media were prepared to 1X and 0.1X concentrations and incubated at 8°C for 1–2 months or RT (20 ± 1°C) for 7–15 days. Bacterial isolates were sub-cultured at least three times onto NA plates and fungi were sub-cultured on PDA plates. The use of 0.1X media and SSMS were to select for “K” strategists.

Following the isolation of pure cultures, genomic DNA was extracted from bacterial isolates using a simplified *FastPrep* bead beating method previously applied for fungal DNA extraction (Ferrari et al., 2011). For all fungal isolates, the Fast DNA® SPIN Kit (MP Biomedicals, NSW Australia) was used following the manufacturer's instructions.

### Bacterial 16S rRNA and fungal internal transcribed spacer (ITS) gene PCR amplification of isolates

All primers required for bacterial and fungal PCR amplification were obtained from Integrated DNA Technology (IDT; MCLeans Ridge, Australia). The reaction components used for bacterial 16S rRNA gene PCR were 10 μl of 5X Go Taq buffer (Promega; Madison, USA), 4 μl MgCl_2_ (25 mM), 1 μl dNTP (10 mM), 40 mM 1 μl each Forward Primer (356F) and Reverse Primer (1064R) (Winsley et al., 2012), 0.25 μl (5 U μl^−1^) Taq polymerase, 2 μl DNA, 5 ml BSA (0.1 μg μl^−1^), 24.75 μl water (Nuclease-free water). The PCR program consisted of an initial denaturation of 95°C for 3 min, 35 cycles of 95°C for 35 s, annealing temperature of 60°C for 30 s and an extension step at 72°C for 5 min.

Fungal internal transcribed spacer (ITS) region genes were amplified using ITS1 forward primer and ITS4 reverse primer (Gardes and Burns, 1993) as described by Ferrari et al. (2011) with slight modification. The PCR reaction mixture consisted of 10 μl of 5X Go Taq buffer, 4 μl MgCl_2_ (25 mM), 1 μl dNTP (10 mM), 40 mM 1 μl each Forward Primer (ITS1) and Reverse Primer (ITS4) (Gardes and Burns, 1993), 0.25 μl (5 U μl^−1^) Taq polymerase, 2 μl DNA, 5 μL BSA (0.1 μg μl^−1^) and 24.75 μl water (Nuclease-free water). The PCR program consisted of a 94°C initial denaturation step for 2 min, followed by 30 cycles of 94°C for 45 s, an annealing temperature of 55°C for 30 s and a final extension step at 72°C for 5 min. PCR amplicons were then visualized on 2% agarose gel stained with SybrSafe (Invitrogen, Paisley UK). The PCR product was treated with two restriction enzymes, *Hinfl* (Promega, Madison, USA) with the cleavage site 5′-G↓ANT C-3′, 3′-C TNA↑G-5′ and *Rsal* (Promega, Madison, USA) with cleavage site 5′↓GTAC-3′-3′ CATG↑ 5′. The reaction component included 0.17 μl restriction enzyme (*Hinfl* or *Rsal*), 2 μl 10X buffer B for *Hinfl* and Buffer C for *Rsal*, 0.2 μl 100X BSA, 2.63 μl water, and 15 μl of PCR product. The mixture was incubated overnight at 37°C and the digest was loaded onto 2% agarose gel and analyzed for band sizes. From selected isolates, PCR products (bacteria and fungi) were then purified by using the QIAquick PCR purification kit from Qiagen following the manufacturer's instructions. The purified PCR amplicons were sequenced at the Ramaciotti Center for Gene Function Analysis (UNSW, Australia) for Sanger sequencing using ABI 3730 Capillary sequencer. The data received were used for isolate identification by blasting (BLASTn) sequences against NCBI database (Johnson et al., 2008).

### Comparison of OTUs recovered using different methods

To compare the OTUs recovered using traditional cultivation and the SSMS from soil samples, the bacterial and fungal OTUs recovered using both cultivation methods were combined with the OTUs identified from total soil community sequencing. For cultured bacteria, the 16S rRNA gene sequences obtained from Sanger sequencing were realigned and trimmed to the same length as the tag sequences obtained from the soil and SSMS enrichments using the MOTHUR pipeline. The common bacterial and fungal OTUs were identified by clustering sequences at 97% identity using USEARCH (Edgar, 2010).

### Data depositing

Bacterial and fungal sequences obtained from the SSMS have been deposited in NCBI under accession number PRJNA350563. For cultured bacterial isolates the sequences were deposited into NCBI under accession numbers KY432693-KY432726, and fungal isolates under KY432734-KY432749 and KY608091. Pyrosequencing data for the soil is available at https://data.aad.gov.au/metadata/records/soil_bacteria_fungi.

## Results

### Microbial diversity of browning peninsula soil

A total of 57,893 16S rRNA and 62,547 ITS quality checked bacterial and fungal reads were recovered after processing the raw datasets from total soil gDNA. Using a similarity threshold of 96% to cluster bacterial sequences and 97% to cluster fungal sequences (Siciliano et al., 2014), a total of 2063 bacterial and 279 fungal OTUs were obtained. Overall bacterial diversity was much greater than fungal diversity (Supplementary Table 1). Rarefaction curves revealed that fungal richness reached asymptote, whilst the bacterial OTU richness was still increasing (Supplementary Figure 1). Soil bacterial diversity detected by tag pyrosequencing spanned 36 phyla, 84 classes, 214 families and 407 genera, whilst fungal diversity was limited to four phyla, spanning 14 classes, 59 families, 104 genera and one unclassified fungi.

Actinobacteria dominated the bacterial communities present, comprising >36% of the total relative abundance across the site. Chloroflexi was the next most abundant phyla, followed by Acidobacteria, Cyanobacteria and Proteobacteria; together accounting for over 54% of total relative abundance within the entire 18 samples analyzed (Supplementary Figure 3). For fungal diversity, Ascomycota dominated all soil samples analyzed, accounting for 93% of the total relative abundance, followed by Basidiomycota (5%) and Zygomycota (1%) (Supplementary Figure 4).

### Microbial diversity following SSMS enrichments and cultivation on artificial media

#### Bacterial diversity

We selected six of the soils from the dataset for a comparison against the SSMS and traditional culturing. In total, 31,797 bacterial quality checked reads representing 401 bacterial OTUs were obtained from the SSMS dataset. Together, the SSMS enrichments recovered 19 phyla (52% of total relative abundance), 61 classes, and 234 (48%) of the genera detected in soil directly. This is 15 times higher than by cultivation only (**Figure 4**), as traditional culturing using artificial media recovered just 4 (11%) of the bacterial phyla and 15 (3%) of the genera originally detected in the soil gDNA sequencing dataset. Despite the large number of quality checked reads for the fungal ITS region, the fungal community, as expected, represented a limited diversity covering just two phyla, 9 classes, and 30 genera.

**Figure 2:**
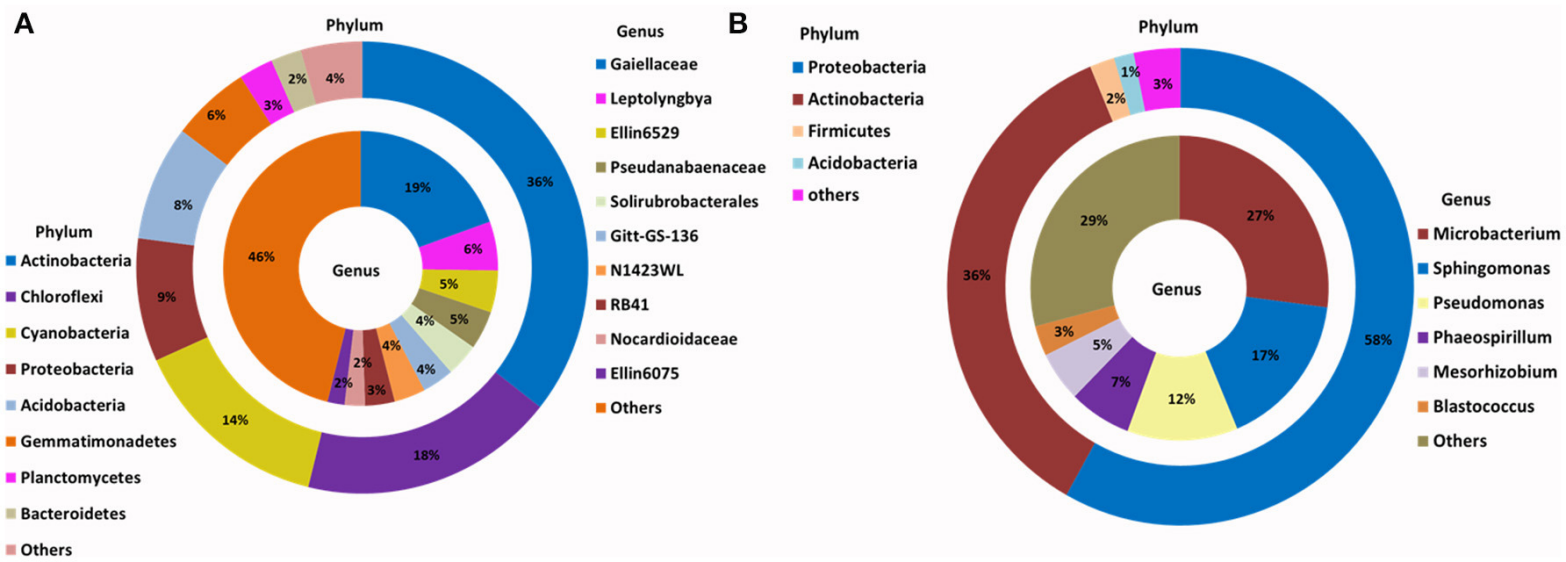
**Phylogenetic distribution of bacteria across (A)** Browning Peninsula soil: data from metabarcoding and **(B)** SSMS soil enrichments: data from metabarcoding. **(A)** Actinobacteria and Chloroflexi were the most dominant bacterial phyla while, unclassified Gaiellaceae and unclassified Ellin6529 were most dominant at the genus level. **(B)** Most abundant bacterial phyla in SSMS enrichments were Proteobacteria followed by Actinobacteria and the dominant genera were *Microbacterium* and *Sphingomonas*. Others represent a sum of relative abundance of <2% phyla or genera present in all soils or SSMS enrichment used.

The relative abundance of major bacterial phyla present in the enriched SSMS varied considerably to the total soil communities (Figure 2). Instead of a dominance of Actinobacteria, Proteobacteria (58%) was most dominant followed by Actinobacteria (36%), Firmicutes (2%), and Acidobacteria (1%). Of major interest was the presence of a further 15 bacterial phyla, such as Bacteroidetes, Chloroflexi, Gemmatimonadetes, TM7, Planctomycetes and Verrucomicrobia representing only 3% of total relative abundance recovered from SSMS enrichments. Of these, Bacteroidetes and Gemmatimonadetes were most dominant; both were present in soil at 2 and 6% of the total relative abundance. There was a 2-fold decrease in the total relative abundance of Bacteroidetes and 6-fold decrease in the total relative abundance of Gemmatimonadetes.

Within Actinobacteria, while the relative abundance was similar between the soil and SSMS enrichments, large shifts in the recovered community were observed at the genus level (Figure 2). For example, unclassified Gaiellaceae, the dominant genera in Browning soil attributing >19% of total relative abundance, was replaced by *Microbacterium* representing 27% of total relative abundance in the SSMS enrichments (Figure 2). While a few reads related to unclassified Gaiellaceae were also retrieved from the SSMS pyrosequencing dataset, the relative abundance was negligible. Other dominant genera in the soil gDNA dataset including Ellin6529 (Chloroflexi; 5%), RB41 (Acidobacteria; 3%), and Solirubrobacterales (Actinobacteria; 4%) were retrieved only as a minor fraction (<0.05%) of the community in the enrichments (Figure 2). The well characterized cyanobacterium, *Leptolyngbya* present at 6% of the relative abundance in the soil dataset was not recovered in the SSMS. Importantly, a further 400 minor genera, for examples *Microbacterium, Sphingomonas, Pseudomonas, Phaeospirillum* and *Blastococcus* which together accounted for just 7% of total relative abundance in soil thrived in the SSMS enrichment reaching up to 67% of the total relative abundance. Among these isolates *Microbacterium, Sphingomonas*, and *Pseudomonas* were endemic to Antarctica.

Following traditional cultivation, 380 bacterial strains were isolated to purity and 207 were successfully extracted and amplified for identification. After screening using RFLP, in total, 34 bacterial strains distributed among 15 bacterial genera within four phyla were identified; Actinobacteria (99–100% identity), Proteobacteria (96–100%), Firmicutes (96–100%) and Bacteroidetes (99% identity) (Table 1). These strains comprised the genera *Kribbella, Mycobacterium, Paenisporosarcina, Sphingomonas, Arthrobacter, Streptomyces*, and *Caulobacter* with >99% identity to known species (Table 1). The most common bacterial isolates matched with *Streptomyces fildesensis* (100% identity) previously isolated from rhizosphere of moss and *Arthrobacter scleromae* (>99% identity) previously isolated from a human specimen. Other bacterial genera isolated were most similar to strains previously isolated from clinical samples, such as *Mycobacterium fluoranthenivorans* (99% identity) and *Dyella japonica* (99% identity).

**Table 1:** **Bacteria recovered from different culture media from Browning Peninsula soils**.

**Strain Name**	**Identification**	**Best match**	**Identity%**	**Accession**	**Phylum**
SPB1	*Arthrobacter phenanthrenivorans*	*Arthrobacter phenanthrenivorans* strain CIM A82	99	KY432693	Actinobacteria
SPB2	*Arthrobacter psychrochitiniphilus*	*Arthrobacter psychrochitiniphilus* strain IARI-R-98	99	KY432694	Actinobacteria
SPB3	*Arthrobacter psychrochitiniphilus*	*Arthrobacter psychrochitiniphilus* strain IARI-R-98	99	KY432695	Actinobacteria
SPB4	*Arthrobacter scleromae*	*Arthrobacter scleromae* strain PAMC 25156	99	KY432696	Actinobacteria
SPB5	*Arthrobacter scleromae*	*Arthrobacter scleromae* strain PAMC 25156	100	KY432697	Actinobacteria
SPB6	*Kribbella ginsengisoli*	*Kribbella ginsengisoli* strain TX1J4	100	KY432698	Actinobacteria
SPB7	*Mycobacterium fluoranthenivorans*	*Mycobacterium fluoranthenivorans* strain S32432	99	KY432699	Actinobacteria
SPB8	*Mycobacterium frederiksbergense*	*Mycobacterium frederiksbergense* strain MQ-200s	99	KY432700	Actinobacteria
SPB9	*Rhodococcus yunnanensis*	*Rhodococcus yunnanensis* strain tibetlhz	100	KY432701	Actinobacteria
SPB10	*Rhodococcus yunnanensis*	*Rhodococcus yunnanensis* strain Tibetlhz-22	100	KY432702	Actinobacteria
SPB11	*Streptomyces beijiangensis*	*Streptomyces beijiangensis* strain TX1G2	100	KY432703	Actinobacteria
SPB12	*Streptomyces fildesensis*	*Streptomyces fildesensis* strain TX1G2	100	KY432704	Actinobacteria
SPB13	*Streptomyces fildesensis*	*Streptomyces fildesensis* strain TX1G2	100	KY432705	Actinobacteria
SPB14	*Streptomyces indigoferus*	*Streptomyces indigoferus* strain 30-4	99	KY432706	Actinobacteria
SPB15	*Streptomyces indigoferus*	*Streptomyces indigoferus* strain 30-4	99	KY432707	Actinobacteria
SPB16	*Streptomyces indigoferus*	*Streptomyces indigoferus* strain 30-4	99	KY432708	Actinobacteria
SPB17	*Streptomyces indigoferus*	*Streptomyces indigoferus* strain 30-4	99	KY432709	Actinobacteria
SPB18	*Streptomyces fildesensis*	*Streptomyces fildesensis* strain TX1G2	100	KY432710	Actinobacteria
SPB19	*Streptomyces fildesensis*	*Streptomyces fildesensis* strain TX1G2	99	KY432711	Actinobacteria
SPB20	*Hymenobacter aerophilus*	*Hymenobacter aerophilus* strain DSM 13606	99	KY432712	Bacteroidetes
SPB21	*Hymenobacter swuensis*	*Hymenobacter swuensis* DY53	99	KY432713	Bacteroidetes
SPB22	*Pedobacter oryzae*	*Pedobacter oryzae* strain NW13	99	KY432714	Bacteroidetes
SPB23	*Paenisporosarcina macmurdoenisis*	*Paenisporosarcina macmurdoenisis* strain WX82	99	KY432715	Firmicutes
SPB24	*Paenisporosarcina macmurdoenisis*	*Paenisporosarcina macmurdoenisis* strain WX82	99	KY432716	Firmicutes
SPB25	*Sporosarcina aquimarina*	*Sporosarcina aquimarina* strain A1-37c-1	99	KY432717	Firmicutes
SP.B26	*Paenisporosarcina quisquiliarum*	*Paenisporosarcina quisquiliarum* strain SK 55	96	KY432718	Firmicutes
SPB27	*Aminobacter aminovorans*	*Aminobacter aminovorans* strain lous 2-3	99	KY432719	Proteobacteria
SPB28	*Caulobacter segnis*	*Caulobacter segnis* ATCC 21756 strain ATCC 21756	99	KY432720	Proteobacteria
SPB29	*Dyella japonica*	*Dyella japonica* strain CRRI-58	99	KY432721	Proteobacteria
SPB30	*Rhizobium phaseoli*	*Rhizobium phaseoli* strain GYS7	100	KY432722	Proteobacteria
SPB31	*Rhizobium qenosp*.	*Rhizobium qenosp*. TUXTLAS-27 strain	100	KY432723	Proteobacteria
SPB32	*Sphingomonas dokdonensis*	*Sphingomonas dokdonensis* strain St15	99	KY432724	Proteobacteria
SPB33	*Sphingopyxis baekryungensis*	*Sphingopyxis baekryungensis*	96	KY432725	Proteobacteria
SPB34	*Sphingomonas faeni*	*Sphingomonas faeni* strain TP-Snow-C72	100	KY432726	Proteobacteria

#### Environmental drivers of bacterial community composition

The measured chemical and physical properties for all 18 soils showed the carbon content across the sampling site ranged from 2.79 to 3.5% w/w (log transformed) while the pH ranged from 6.38 to 6.75 (Supplementary Table 2). It was expected that the distribution of frost boils across the Browning Peninsula would influence the localized soil chemical and physical environment, and subsequently the microbial diversity distribution. This was found to be the case when tested with an analysis of similarity (ANOSIM), with the frost boils significantly correlated to both the local soil physical and chemical parameters (*P* < 0.001, a global *R*-value of 0.726) and the distribution of the bacterial community (*P* < 0.001, a global *R*-value of 0.922). However, the frost boil distribution had no significant correlation with the fungal community composition (*P* > 0.1, a global *R*-value of −0.215).

In this study, the environmental factors chlorine, phosphate, aspect and elevation were observed to be significantly affecting bacterial community distribution across the site. However, fungal distributions were not affected by any of the environmental variables analyzed. Chlorine content was observed to be most significant predictor variable with pseudo *F* = 3.75, *P* < 0.001, and the biological variance of 19% (**Table 3**). Furthermore, aspect degree, phosphate and elevation added up to 25% of the biological variance. For other variables, such as soil conductivity (18%), grain size (sand and mud together 21%), and total nitrogen (9%), the variables were only statistically significant when analyzed individually (**Table 3**).

#### Fungal diversity

**Figure 3:**
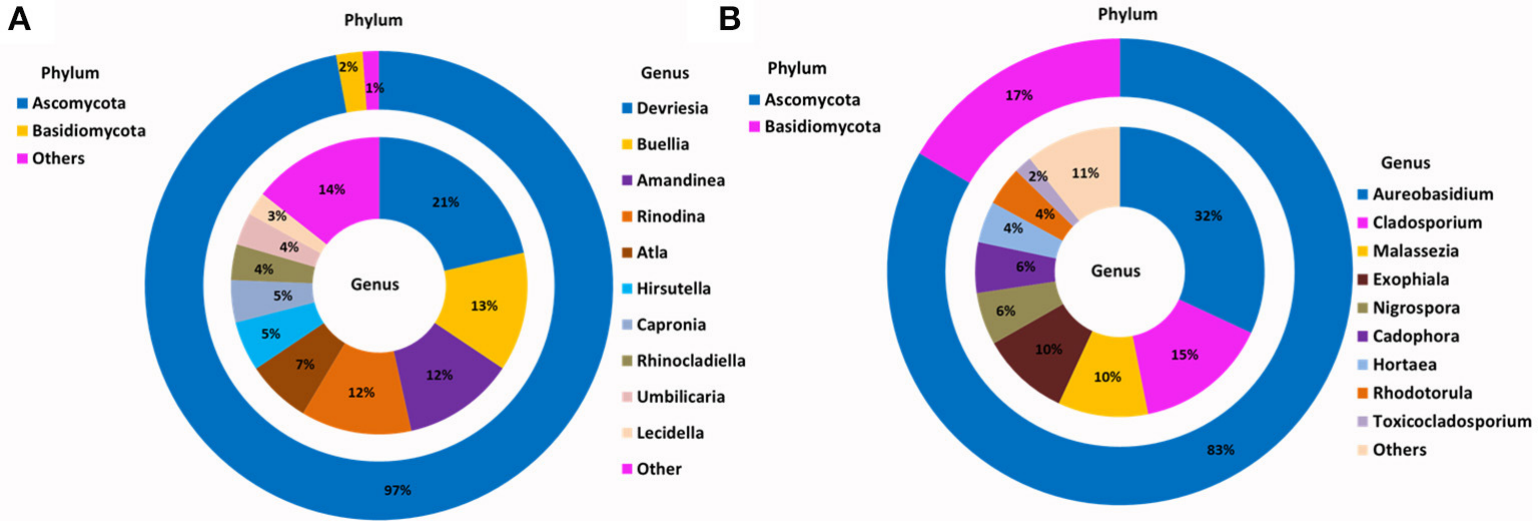
**Phylogenetic distribution of fungi across (A)** Browning Peninsula soil: data from metabarcoding and **(B)** SSMS soil enrichments: data from metabarcoding. **(A)** Dominant fungal phyla in Browning Peninsula soil were Ascomycota followed by Basidiomycota and at the genera level *Devriesia* and *Buellia* were most abundant. **(B)** Only Ascomycota and Basidiomycota were enriched in the SSMS, with *Aureobasidium* the most dominant fungal genera recovered. Others represent a sum of <2% relative abundance of other phyla or genera present in the soils or SSMS enrichments.

For the SSMS enrichments, 110,851 fungal quality checked reads representing 86 fungal OTUs were obtained following tag pyrosequencing. It was demonstrated here that in addition to bacteria various fungi, particularly microfungi (*Cladosporium, Malassezia, Exophiala, Horteae*, and *Rhodotorula*) were successfully enriched for using the SSMS (Figure 3). While three phyla dominated the soil dataset, following SSMS enrichments, only two were remained with the relative abundance of Basidiomycota increasing from 2 to 17% (Figure 3).

At the genus level, *Devriesia* (Ascomycota), which represented >30% of total relative abundance of soils, as well as *Buellia* (Ascomycota) which represented >13% of relative abundance and *Amandinae, Capronia, Rinodina, Atla*, and *Hirsutella* (together representing 41% of relative abundance) were not detected in the enrichments. Instead, *Aureobasidium* (Ascomycota; 32%) appeared as the most dominant fungi in the SSMS enrichment. As with bacteria, 96 minor genera present in the soil gDNA dataset, which accounted for just 14% of the total relative abundance (ie. *Cladosporium, Malassezia* and *Rhodotorula*), were selected for in the SSMS enrichments, reaching up to 29% of the total relative abundance (Figure 3). Among these *Malassezia* and *Rhodotorula* belonged to the Basidiomycota.

Using artificial media, 80 fungal strains were isolated to purity. After RFLP, 17 strains were selected for sequencing. In total, 17 strains spanning two phyla, Ascomycota (>96% identity) and Basidiomycota (>98% identity), were identified including *Cryptococcus, Phoma, Engyodontium, Thelobolus and Cladosporium* (Table 2). Almost all fungal strains isolated from Browning Peninsula were microfungi with >98% similarity to previously isolated fungi species (Table 2). The most abundant microfungi strain recovered belonged to *Geomyces pannorum* (99% identity), previously isolated from the cyst of a nematode (*Globodera pallida*) and *Holtermanniella watticus* (>99% identity), previously isolated from King George Island, Chile (Table 2).

**Table 2:** **Fungi recovered from different cultivation media from Browning Peninsula soil**.

**Strain Name**	**Isolate**	**Closest Match**	**Identity (%)**	**Accession**	**Phylum**
SPF1	*Geomyces pannorum*	*Geomyces pannorum* isolate E10	99	KY432734	Ascomycota
SPF2	*Thelobolus microsporous*	*Thelobolus microsporous*	99	KY432735	Ascomycota
SPF3	*Thelobolus microsporous*	*Thelobolus microsporous* isolate10 BI	95	KY432736	Ascomycota
SPF4	*Pseudeurotium bakeri*	*Pseudeurotium bakeri* strain 842	95	KY432737	Ascomycota
SPF5	*Cladosporium grevilleae*	*Cladosporium grevilleae* strain CBS 114271	99	KY432738	Ascomycota
SPF6	*Phoma herbarum*	*Phoma herbarum* CBS 615.75	99	KY432739	Ascomycota
SPF7	*Thelobolous globosus*	*Thelobolous globosus* isolate ANT03-221	100	KY432740	Ascomycota
SPF8	*Chaetomium globosum*	*Chaetomium globosum* isolate TNAU Cq	99	KY432741	Ascomycota
SPF9	*Engyodontium album*	*Engyodontium album* strain LVPEI.H1584	99	KY432742	Ascomycota
SPF10	*Cladosporium cladosporides*	*Cladosporium cladosporides* strain DUCC5020	99	KY432743	Ascomycota
SPF11	*Geomyces pannorum*	*Geomyces pannorum* isolate E10	99	KY432744	Ascomycota
SPF12	*Cladosporium oxysporum*	*Cladosporium oxysporum* strain CASVK1	99	KY432745	Ascomycota
SPF13	*Thelobolus globosus*	*Thelobolus globosus* strain UFMCB 6095	96	KY432746	Ascomycota
SPF14	*Cryptococcus victoriae*	*Cryptococcus victoriae* strain P41A001	98	KY432747	Basidiomycota
SPF15	*Peniophora lycii*	*Peniophora lycii*	99	KY608091	Basidiomycota
SPF16	*Holtermanniella watticus*	*Holtermanniella watticus* isolate T2Hw	99	KY432748	Basidiomycota
SPF17	*Holtermanniella watticus*	*Holtermanniella watticus*	100	KY432749	Basidiomycota

### Comparison of culture-dependent and -independent techniques

**Figure 4:**
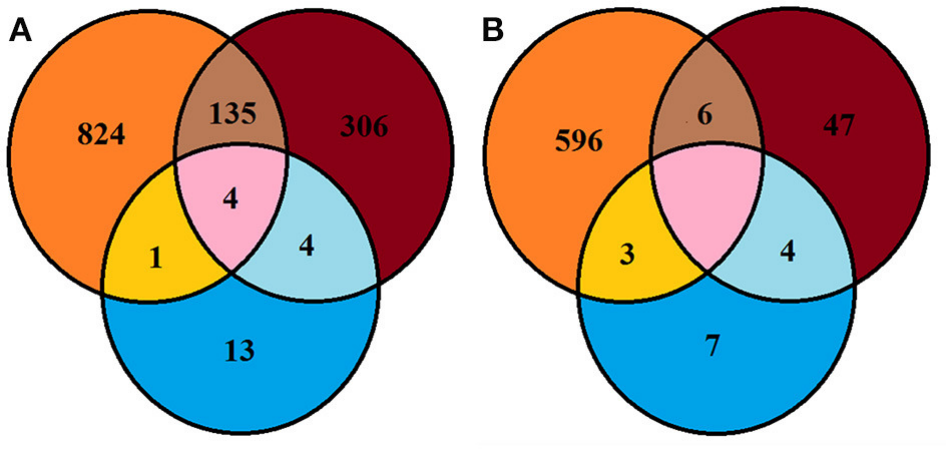
**Venn diagram representing Browning Peninsula microbial diversity at the OTU level recovered from all three methods used in this study (A)** bacteria **(B)** fungi. Orange circles indicate OTUs recovered from the soil datasets, blue represents the recovery of OTUs from artificial cultivation and brown represents OTUs recovered from the SSMS. The pink area represents common OTUs recovered from the soil, SSMS microcultivation and artificial cultivation.

A large proportion of bacterial and fungal OTUs were not shared between culture dependent and independent techniques (Figure 4). For example, 135 OTUs detected were common in soil and SSMS datasets; only four OTUs were common in SSMS and artificial media and one OTU was present in the artificial media and soil datasets (Figure 4). In fact, there were more unique bacterial genera retrieved than shared between techniques. For instance, 824 bacterial OTUs were not retrieved from culture-dependent techniques. Overall, only 13 unique OTUs were obtained from artificial-cultivation. Surprisingly, 306 bacterial OTUs recovered from SSMS were also not detected in the other two approaches suggesting minor taxa are enriched for using the SSMS (Figure 4).

The Shannon diversity index for fungi retrieved from the soil gDNA dataset was 3.51 markedly lower than for bacteria (5.5) (Supplementary Table 1). A similar recovery pattern of bacterial and fungal communities was observed from the SSMS enrichments with a Shannon index of 3.15 for bacteria and 2.57 for fungi. Combined, a total of 663 fungal OTUs were recovered using three approaches, yet no common OTU was recovered from all three methods used. Altogether, six of the fungal OTUs recovered were common in the soil and SSMS enrichments, four OTUs were common in enrichments and artificial media, while three OTUs were present in the soil and artificial media datasets. Similar to the bacterial data, 596 unique fungal OTUs were retrieved from the soil dataset only, 47 OTUs from SSMS enrichments and seven OTUs from cultivation (Figure 4).

Comparing recovery of individual phylum level OTUs from both soil and SSMS, SSMS recovered 39.5% of unique Proteobacteria, 23% of Actinobacteria, 27% of Bacteroidetes and 24% of Firmicutes (Supplementary Table 4). The shared OTUs were 10% for Proteobacteria, 11% for Actinobacteria, 12% for Bacteroidetes and 3.5% for Firmicutes. The recovery of fungal unique Ascomycota OTUs from soil was 90% and SSMS was 9%. For Basidiomycota unique OTUs obtained were 87% from soil and 7.23% from SSMS (Supplementary Table 5).

## Discussion

### Microbial diversity and distribution across browning peninsula

The soils of Browning Peninsula harbor a bacterial community dominated by Actinobacteria (up to a 36% relative abundance), followed by Chloroflexi, Cyanobacteria, Proteobacteria and Acidobacteria. The high abundance of Actinobacteria is in stark contrast to Arctic and other Antarctic sites commonly dominated by Proteobacteria, Cyanobacteria, and Acidobacteria (Steven et al., 2007, 2013; Koyama et al., 2014). For example, the McMurdo Dry Valleys, a well-studied site of Antarctica, has been found to be dominated by Proteobacteria (Clocksin et al., 2007; Cary et al., 2010); Miers Valley, is dominated by Bacteriodetes, Proteobacteria, and Actinobacteria (Stomeo et al., 2012), and Victoria Land is comprised of a heterogeneous bacterial community encompassing Proteobacteria, Acidobacteria, Actinobacteria, Chloroflexi, and Cyanobacteria (Kim et al., 2015). The variability in diversity structures in each of these sites may be due to variable organic matter content (Takebayashi et al., 2007; Bell et al., 2013; Ji et al., 2015). Previously, the dominance of Proteobacteria was linked with areas rich in higher organic matter (Bell et al., 2013) and moisture content (Horn et al., 2014; Niederberger et al., 2015), while dominance of Actinobacteria has been documented in arid soils (Niederberger et al., 2015), and linked to low moisture and nutrients (Takebayashi et al., 2007). Further biotic and abiotic factors, such as plants, penguins, birds, seal carcasses, and human activity are also reported to affect microbial community distributions across Antarctica (Cowan et al., 2014; Wang et al., 2015).

Although fungi are more tolerant of freezing and thawing compared to bacteria (Sharma et al., 2006), the fungal diversity of Browning soil was markedly lower than that of the bacterial community. Of those fungi present, Browning soil was dominated by Ascomycota (>97% of the total relative abundance) and was limited to four phyla only. In contrast, nearby Eastern Antarctic sites that do not contain frost boils harbor at least 60% more fungal richness than Browning Peninsula (Ferrari et al., 2015). A lack of tolerance of extreme variation in the salinity, combined with continuous mixing of soil due to cryoturbation, may have limited the fungal diversity at Browning Peninsula (Ferrari et al., 2015). Dispersal limitation across the site due to the presence of hard-edge barriers surrounding frost boils, may also contribute to the reduced fungal richness observed compared to highly connected Antarctic sites, as the larger size of fungi limits their dispersal compared to bacteria (Rao et al., 2011; Ferrari et al., 2015). Moreover, the lack of vegetation (Stewart et al., 2011), and fungal vectors including insects and animals could contribute to reduced fungal diversity in Antarctica compared with those of the Arctic. Fungi has been shown to have symbiotic association with plants (Geml et al., 2012; Timling et al., 2012) and no plant life has been documented at Browning Peninsula (Stewart et al., 2011).

### Environmental drivers of microbial community

The environmental factors chlorine, phosphate, aspect and elevation were observed to affect the bacterial community at Browning, whilst none of the analyzed environmental parameters were observed to be statistically significant to affect fungal community variation. Of the environmental parameters analyzed, chlorine was primarily correlated to the bacterial community composition, contributing 19% of the total biological variance. Chlorine was not significant to fungal community variation here, but has been previously observed to have strong effects on polar fungi (Siciliano et al., 2014). Elevation was also observed to affect the bacterial distribution and several in-depth studies of effect of elevation on microbial community have revealed dramatic shifts in the microbial taxa along elevation transects (Bryant et al., 2008; Cowan et al., 2010; Shen et al., 2013). It is also well established that the soil heterogeneity of Arctic and Antarctic soil depends on organic matter (total carbon) and nitrate (total nitrogen) availability (Michaelson et al., 2012; Shen et al., 2013; Siciliano et al., 2014). The reduced bacterial and fungal diversity of Browning Peninsula soil may, therefore, be a result of the relatively poor nutrient content of these soils (Chong et al., 2009). The phosphate content and aspect were also correlated to the bacterial community contributing to 19% of the total variation (Table 3). In contrast, fungal diversity distributions have been previously linked with pH (Connell et al., 2006; Arenz and Blanchette, 2011), conductivity (Arenz and Blanchette, 2011) and moisture content (Connell et al., 2006). However, none of the environmental factors were significantly correlated with the fungal community distribution here, which may be due to the unique landscape and limited diversity present.

**Table 3:** **DistLM results indicating the correlation between environmental parameters as a predictor of the microbial community distribution**.

**Environmental variables**	**Marginal test**			**Sequential tests**			
	**Pseudo-*F***	***P***	**Prop**.	**Pseudo-*F***	***P***	**Prop**.	**^§^Cumul**
Chlorine	3.75	**0.001**	0.19	3.757	**0.001**	0.19	0.190
Aspect^a^	2.84	**0.002**	0.15	2.65	**0.001**	0.12	0.31
Phosphate	2.002	**0.011**	0.011	1.59	**0.014**	0.07	0.38
Elevation^a^	2.20	**0.014**	0.12	1.46	**0.028**	0.062	0.44
SO_4_	2.30	**0.007**	0.12	1.32	0.099	0.55	0.50
Mud% <63 μm	1.97	**0.018**	0.11	1.38	0.082	0.05	0.55
Sand	2.11	**0.01**	0.11	1.23	0.217	0.04	0.60

Marginal test results are based on individual predicator variables. Sequential test results are based on the relative proportion when all variables are considered. P < 0.05 was considered as significant. Pseudo-F represented a multivariate version of fisher's F statistic and

§ *Cumul represented the cumulative proportion of variance. All values are in mg kg^−1^ unless specified*.

a *represent value degree. P values were considered significant at < 0.05 and are represented in bold*.

### Novel cultivation approaches to enrich polar soil microbes

The present investigation was the first study to culture fungi using the SSMS, with 30 non-lichenized microfungi including *Toxicocladosporium, Exophiala, Hortaea*, and *Cadophora* species selected for using the approach. From the two phyla recovered, Ascomycota members enriched for included *Aureobasidium, Cladosporium, Nigrosphora* and *Cadophora*, while Basidiomycota members included *Malassezia, Hortaea*, and *Rhodotorula* (Figure 3). Most of these fungal genera have been previously isolated from soils, plants and animals. However, the SSMS appeared to facilitate the cultivation of a wide range of microfungi, as demonstrated by the recovery of the endemic mesophilic psychrotolerant microfungi, such as *Aureobasidium* and *Cladosporium* (Zucconi et al., 2011) to host specific genera, such as *Nigrosphora, Malassezia* genera; a plant and animal pathogen (Hudson, 1963; Velegraki et al., 2015), *Cadophora*; a “soft rot” wood decomposer isolated from Antarctic hut wood (Blanchette et al., 2004; Ludley and Robinson, 2008), *Rhodotorula*; previously isolated from Windmill Island, Antarctica soils (Gesheva, 2010) and *Hortaea*; a halotolerant black yeast (Plemenitas et al., 2008). For bacteria, the SSMS enrichments led to an increase in Proteobacteria relative abundances up to 58%, highlighting the system's selective bias toward heterotrophs (Ferrari et al., 2008). Under the conditions used, the SSMS failed to enrich for Cyanobacteria, Chlorobi and Chloroflexi. Further modifications to include a light source may aid in the isolation of these phototrophic taxa.

The most commonly isolated bacteria *Arthrobacter* and *Streptomyces* were previously isolated from Antarctic cold deserts (Smith et al., 2006), post-glacial soils (Zdanowski et al., 2012) and Princess Elisabeth Station soil (Peeters et al., 2011), temperate soils and variety of other sources (Kaewkla and Franco, 2013). For fungal isolates, *Geomyces, Thelobolus* and *Phoma* have been previously recovered from Antarctic soils (Frate and Caretta, 1990; Azmi and Seppelt, 1998; Arenz et al., 2006; Connell et al., 2006; Arenz and Blanchette, 2011; Godinho et al., 2013). Interestingly, *Chrysosporium* sp., *Mortierella gamsii, Mycelia sterilia, Phoma* sp., and *Thelobolus microspores* have also been previously isolated from Browning Peninsula (Azmi and Seppelt, 1998). Similar to *Devriesia* the requirement for a heat pre-treatment to germinate cells before culturing into artificial media (Onofri et al., 2011) may have been a limiting factor in the diversity of cultured members. The recovery of cold-adapted bacterial strains, such as *Dyella* have been proposed to degrade single molecules, such as N-Acylhomoserine Lactones (Chen and Chan, 2012), and UV radiation-resistant fungi, such as *Phoma* (Hughes et al., 2003) may have significant biotechnological potential that warrants further investigation.

### Use of molecular and cultivation approaches as a better representation of bacterial and fungal diversity

Of the 1284 bacterial OTUs and 663 fungal OTUs detected using multiple approaches here, tag pyrosequencing from soil gDNA detected 75% of the total bacterial and 91% of total fungal community diversity, which is in line with previous studies reporting ≤60% of the total bacterial diversity detected using sequencing alone (Vaz-Moreira et al., 2011; Stefani et al., 2015). It is well established that very low percentages of bacteria have been recovered into pure culture (Handelsman, 2004; Ferrari et al., 2008), and here 1.7% of the total bacteria diversity was recovered into artificial media. This recovery increased to 34% within the SSMS enrichments, highlighting the potential of this approach to recover novel species (van Dorst et al., 2016). This is comparable to the recovery of 50% of total bacterial diversity using the ichip method (a similar culturing technique) described by Nichols et al. (2010).

Only four of the total bacterial OTUs detected were present in all three techniques used. The absence of consistency between techniques, or a “culture clash” has been consistently observed between clone libraries and cultivation attempts (Maturrano et al., 2006). Previously, higher community diversity (2.4% of bacterial and 8.2% of fungal communities) has been shared between artificial media and tag pyrosequencing from hydrocarbon contaminated soils(Stefani et al., 2015; van Dorst et al., 2016). The rapid growth of microfungi, particularly yeasts on the SSMS may have occurred following the addition of water to the soils (Vishniac, 1996) with freely available water potentially aiding the germination of spores.

Microfungi are difficult to culture and enrichment of microfungi using the SSMS offers a new opportunity to harness the biotechnological potential of cold-adapted Antarctic fungi. Despite inherent bias associated with the techniques used, such as selection of phenotypic characteristics of colonies from artificial media (Tytgat et al., 2014), DNA extraction (Evans and Seviour, 2012; Plassart et al., 2012), PCR amplification (Schloss et al., 2011) and inadequate sequence-depth when carrying out 454 tag pyrosequencing (Kim et al., 2011; Kumar et al., 2011; Lagier et al., 2012), multiple approaches delivered a comprehensive view of the soil microbial community of Browning Peninsula, unveiling an ecosystem dominated by rare unclassified bacterial taxa combine with extremely low fungal diversity.

## Author contributions

All authors contributed to the study design. SP, MJ, JV, and AP carried out the experimental procedure and data analysis of the present study. SP prepared the draft of the manuscript. SP, JW, MJ, JV, IS, AP, and BF finalized the manuscript. All authors read and approved the manuscript. JW and BF coordinated this study.

### Conflict of interest statement

The authors declare that the research was conducted in the absence of any commercial or financial relationships that could be construed as a potential conflict of interest.
