# Supplementary material for: Microbial Diversity of Browning Peninsula, Eastern Antarctica Revealed Using Molecular and Cultivation Methods
Source: Front Microbiol. 2017 Apr 7;8:591. doi: 10.3389/fmicb.2017.00591 (PMC5383709; doi:10.3389/fmicb.2017.00591)
Supplement: Supplementary file 1 [file Table1.PDF]

Supplementary Material

Microbial Diversity of Browning Peninsula, Eastern Antarctica Revealed using Molecular and Cultivation Methods

Sarita Pudasaini[1], John Wilson[1], Mukan Ji[1], Josie van Dorst[1], Ian Snape[2], Anne S. Palmer[2], Brendan P. Burns[1] and Belinda C. Ferrari[1*]

[1]School of Biotechnology and Biomolecular Sciences, UNSW Sydney, Kensington, New South Wales, Australia, 2052

[2]Australian Antarctic Division, Department of Sustainability, Environment, Water, Population and Communities, Kingston, Tasmania, Australia, 7050

* **Correspondence**: Dr. Belinda C. Ferrari, School of Biotechnology and Biomolecular Sciences, UNSW Australia, 2052. Phone: (+61 2) 9385 2032. Fax: (+61 2) 9385 1483. Email: b.ferrari@unsw.edu.au

Supplementary Tables

Supplementary Table 1. Calculated diversity estimates for bacteria and fungi.

454 sequencing	Unique OTUs	Total reads	Margalef's Diversity index (M)	Pielou's Evenness index (J')	Shannon Diversity index H' (loge)	1-Simpson
Soil bacteria	962	57839	188.04	0.72103	5.5028	0.98421
SSMS bacteria	449	31797	38.487	0.52638	3.15541	0.88317
Soil fungi	605	62547	34.68	0.591	3.5170	0.8944
SSMS fungi	57	110851	7.317	0.5780	2.5747	0.86375
