# Supplementary material for: Microbial Diversity of Browning Peninsula, Eastern Antarctica Revealed Using Molecular and Cultivation Methods
Source: Front Microbiol. 2017 Apr 7;8:591. doi: 10.3389/fmicb.2017.00591 (PMC5383709; doi:10.3389/fmicb.2017.00591)
Supplement: Supplementary file 2 [file Table2.PDF]

Supplementary Material

Microbial Diversity of Browning Peninsula, Eastern Antarctica Revealed using Molecular and Cultivation Methods

Sarita Pudasaini[1], John Wilson[1], Mukan Ji[1], Josie van Dorst[1], Ian Snape[2], Anne S. Palmer[2], Brendan P. Burns[1] and Belinda C. Ferrari[1*]

[1]School of Biotechnology and Biomolecular Sciences, UNSW Sydney, Kensington, New South Wales, Australia, 2052

[2]Australian Antarctic Division, Department of Sustainability, Environment, Water, Population and Communities, Kingston, Tasmania, Australia, 7050

* **Correspondence**: Dr. Belinda C. Ferrari, School of Biotechnology and Biomolecular Sciences, UNSW Australia, 2052. Phone: (+61 2) 9385 2032. Fax: (+61 2) 9385 1483. Email: b.ferrari@unsw.edu.au

Supplementary Tables

Supplementary Table 2. Environmental data: chemical variables of Browning Peninsula soils.

Samples	Chemical Parameters								
	*Cl	*NO_3	*SO_4	*NH_4	*PO_4	*TKN	*TKP	€TC	pH
BP1	3.26	0.98	2.05	0.59	6.06	-1.56	-1.2	3.5	6.66
BP2	4.01	1.02	2.32	-0.98	5.19	-1.82	-2.12	3.1	6.7
BP3	3.6	0.33	2.25	0.23	4.7	-1.82	-2.12	2.79	6.64
BP4	4.08	0.09	2.47	0.09	5.14	-1.61	-1.43	3.22	6.58
BP5	3.4	1.48	1.98	-0.08	5.39	-1.65	-1.35	3.06	6.4
BP6	2.61	-0.05	1.5	0.16	5.01	-1.38	-1.61	2.88	6.6
BP7	3.61	1.04	2.4	0.24	5.14	-1.55	-1.83	3.17	6.75
BP8	3.68	-0.02	2.36	-0.12	5.25	-1.52	-1.77	3.22	6.64
BP9	4.06	0.91	2.4	-0.98	4.5	-1.84	-2.21	3.06	6.46
BP10	3.11	-0.14	1.43	-0.98	4.5	-1.59	-2.41	3.03	6.52
BP11	3.15	0.59	2.17	0.19	5.67	-1.59	-1.56	2.99	6.7
BP12	4.37	0.75	2.41	0.51	5.19	-1.6	-1.43	3.04	6.53
BP13	4.09	0.24	2.29	-0.06	5.25	-1.62	-1.39	3.08	6.38
BP14	2.26	-0.1	0.97	0.23	4.94	-1.59	-1.83	2.95	6.54
BP15	2.63	0.8	1.71	1.03	5.52	-1.6	-0.92	3.05	6.46
BP16	4.18	-0.11	2.47	0.12	4.79	-1.59	-1.56	2.93	6.52
BP17	2.18	0.77	1.45	0.6	5.35	-1.58	-1.27	2.96	6.51
BP18	3.05	0.93	2.14	0.27	5.6	-1.48	-2.04	3.38	6.59

*Cl= Log(Cl (mg kg^{-1} DMB)), *NO_2= Log(NO_2 (mg kg^{-1}, DMB)), *NO_3= Log(NO_3 (mg kg^{-1}, DMB)), *SO_4 = Log(SO_4 (mg kg^{-1} DMB)), *NH_4=Log(NH_4 (mg kg^{-1} DMB)), * PO_4 =

Log(PO_4 (mg kg^{-1} DMB)), *TKN = Log(TKN (mg kg^{-1} DMB)), * TKP = Log(TKP (mg kg^{-1} DMB)), [€]TC = Log(TC (% w/w))
