# Supplementary material for: Microbial Diversity of Browning Peninsula, Eastern Antarctica Revealed Using Molecular and Cultivation Methods
Source: Front Microbiol. 2017 Apr 7;8:591. doi: 10.3389/fmicb.2017.00591 (PMC5383709; doi:10.3389/fmicb.2017.00591)
Supplement: Supplementary file 3 [file Table3.PDF]

Supplementary Material

Microbial Diversity of Browning Peninsula, Eastern Antarctica Revealed using Molecular and Cultivation Methods

Sarita Pudasaini[1], John Wilson[1], Mukan Ji[1], Josie van Dorst[1], Ian Snape[2], Anne S. Palmer[2], Brendan P. Burns[1] and Belinda C. Ferrari[1*]

[1]School of Biotechnology and Biomolecular Sciences, UNSW Sydney, Kensington, New South Wales, Australia, 2052

[2]Australian Antarctic Division, Department of Sustainability, Environment, Water, Population and Communities, Kingston, Tasmania, Australia, 7050

* **Correspondence**: Dr. Belinda C. Ferrari, School of Biotechnology and Biomolecular Sciences, UNSW Australia, 2052. Phone: (+61 2) 9385 2032. Fax: (+61 2) 9385 1483. Email: b.ferrari@unsw.edu.au

Supplementary Tables

Supplementary Table 3. Environmental data: physical variables of Browning Peninsula soils

Soil Samples	Physical parameters						
	Ele	Aspect	´Slope	*Con	¥Mud	¤Sand	§Gra
BP1	3.7	293.85	1.43	3.38	1.49	63.66	31.44
BP2	3.69	80.14	1.16	3.88	1.41	69.77	26.26
BP3	3.7	293.36	1.4	3.6	1.96	70.25	15.1
BP4	3.69	90.41	1.08	3.92	1.47	82.84	12.47
BP5	3.77	127.34	0.28	3.43	1.27	68.09	29.34
BP6	3.77	143.87	0.28	2.79	1.99	58.06	26.24
BP7	3.7	295.74	1.38	3.61	1.37	67.86	28.57
BP8	3.7	297.32	1.34	3.72	1.48	78.7	16.46
BP9	3.69	83.61	1.13	3.88	1.37	70.67	25.84
BP10	3.77	123.98	0.33	3.46	1.64	73.09	19.61
BP11	3.7	294.88	1.38	3.48	1.57	72.71	21.23
BP12	3.69	85.4	1.11	4.31	1.31	76.76	20.31
BP13	3.69	88.9	1.09	3.92	1.46	78.06	17.44
BP14	3.77	140.3	0.3	2.54	1.41	66.01	30.04
BP15	3.77	136.53	0.3	2.98	1.53	69.96	24.55
BP16	3.69	93.93	1.06	4.03	1.47	75.12	20.27
BP17	3.77	147.55	0.31	2.77	1.52	71.7	22.94
BP18	3.7	292.62	1.44	3.3	1.53	65.4	29.1

*Ele= Elevation degree, Con = Log(conductivity (uS/cm)), ¥Mud =<63 µm, ¤Sand = % Sand 63-2000 µm, §Gra=% Gravel >2mm, ´Slope= Log(Slope (deg)).
