# Supplementary material for: Microbial Diversity of Browning Peninsula, Eastern Antarctica Revealed Using Molecular and Cultivation Methods
Source: Front Microbiol. 2017 Apr 7;8:591. doi: 10.3389/fmicb.2017.00591 (PMC5383709; doi:10.3389/fmicb.2017.00591)
Supplement: Supplementary file 4 [file Table4.PDF]

## *Supplementary Material*

# Microbial Diversity of Browning Peninsula, Eastern Antarctica Revealed using Molecular and Cultivation Methods

**Sarita Pudasaini[1], John Wilson[1], Mukan Ji[1], Josie van Dorst[1], Ian Snape[2], Anne S. Palmer[2], Brendan P. Burns[1] and Belinda C. Ferrari[1*]**

[1]School of Biotechnology and Biomolecular Sciences, UNSW Sydney, Kensington, New South Wales, Australia, 2052

[2]Australian Antarctic Division, Department of Sustainability, Environment, Water, Population and Communities, Kingston, Tasmania, Australia, 7050

* **Correspondence**: Dr. Belinda C. Ferrari, School of Biotechnology and Biomolecular Sciences, UNSW Australia, 2052. Phone: (+61 2) 9385 2032. Fax: (+61 2) 9385 1483. Email: b.ferrari@unsw.edu.au

## Supplementary Tables

**Supplementary Table 4**: The OTU abundance table of top 10 bacterial phyla present after clustering the Soil and SSMS dataset.

| Phylum level | Soil OTUs | SSMS OTUs | Shared OTUs | Total OTUs | Unique OTUs (%) | | Shared OTUs (%) |
|---|---|---|---|---|---|---|---|
| | | | | | Soil | SSMS | |
| Proteobacteria | 205 | 170 | 36 | 339 | 49.85 | 39.53 | 10.62 |
| Actinobacteria | 187 | 84 | 27 | 244 | 65.57 | 23.36 | 11.07 |
| Chloroflexi | 100 | 22 | 12 | 110 | 80.00 | 9.09 | 10.91 |
| Planctomycetes | 78 | 14 | 7 | 85 | 83.53 | 8.24 | 8.24 |
| Bacteroidetes | 75 | 41 | 13 | 103 | 60.19 | 27.18 | 12.62 |
| Acidobacteria | 67 | 45 | 20 | 92 | 51.09 | 27.17 | 21.74 |
| Cyanobacteria | 45 | 3 | 0 | 48 | 93.75 | 6.25 | 0.00 |
| Firmicutes | 43 | 16 | 2 | 57 | 71.93 | 24.56 | 3.51 |
| Verrucomicrobia | 41 | 17 | 7 | 51 | 66.67 | 19.61 | 13.73 |
| Gemmatimonadetes | 39 | 13 | 8 | 44 | 70.45 | 11.36 | 18.18 |
