# Supplementary material for: Microbial Diversity of Browning Peninsula, Eastern Antarctica Revealed Using Molecular and Cultivation Methods
Source: Front Microbiol. 2017 Apr 7;8:591. doi: 10.3389/fmicb.2017.00591 (PMC5383709; doi:10.3389/fmicb.2017.00591)
Supplement: Supplementary file 5 [file Table5.PDF]

Supplementary Material

Microbial Diversity of Browning Peninsula, Eastern Antarctica Revealed using Molecular and Cultivation Methods

Sarita Pudasaini[1], John Wilson[1], Mukan Ji[1], Josie van Dorst[1], Ian Snape[2], Anne S. Palmer[2], Brendan P. Burns[1] and Belinda C. Ferrari[1*]

[1]School of Biotechnology and Biomolecular Sciences, UNSW Sydney, Kensington, New South Wales, Australia, 2052

[2]Australian Antarctic Division, Department of Sustainability, Environment, Water, Population and Communities, Kingston, Tasmania, Australia, 7050

* **Correspondence**: Dr. Belinda C. Ferrari, School of Biotechnology and Biomolecular Sciences, UNSW Australia, 2052. Phone: (+61 2) 9385 2032. Fax: (+61 2) 9385 1483. Email: b.ferrari@unsw.edu.au

Supplementary Tables

Supplementary Table 5: The OTU abundance table within each fungal phyla present after clustering the Soil and SSMS dataset.

Phylum level	Soil OTUs	SSMS OTUs	Shared OTUs	Total OTUs	Unique OTUs (%)		Shared OTUs (%)
					Soil	SSMS	
Ascomycota	451	47	2	496	90.52	9.07	0.40
Basidiomycota	77	10	4	83	87.95	7.23	4.82
Chytridiomycota	48	0	0	48	100.00	0.00	0.00
Zygomycota	28	0	0	28	100.00	0.00	0.00
unclassified_Fungi	1	0	0	1	100.00	0.00	0.00
